# Transcranial Random Noise Stimulation over the Dorsolateral Prefrontal Cortex in Patients with Parkinson’s Disease and Mild Cognitive Impairment: A Pilot Study

**DOI:** 10.3390/brainsci15111232

**Published:** 2025-11-16

**Authors:** Davide Mazzara, Angelo Torrente, Paolo Alonge, Roberta Baschi, Marina Campione, Vincenzo Di Stefano, Giuseppe La Bianca, Filippo Brighina, Roberto Monastero

**Affiliations:** 1 Department of Biomedicine, Neuroscience and Advanced Diagnostics (Bi.N.D.), University of Palermo, 90127 Palermo, Italy; davide.mazzara29@gmail.com (D.M.); angelo.torrente@unipa.it (A.T.); paolo.alonge01@unipa.it (P.A.); roberta.baschi@gmail.com (R.B.); vincenzo.distefano07@unipa.it (V.D.S.); giuseppelabianca92@gmail.com (G.L.B.); filippo.brighina@unipa.it (F.B.); 2 Department of Neurology, A.R.N.A.S. Civico Di Cristina Benfratelli Hospital, 90127 Palermo, Italy; 3 Faculty of Economics and Law, University of Enna Kore, 94100 Enna, Italy; marina.campione@unikorestudent.it; 4 Department of Oncology, A.R.N.A.S. Civico Di Cristina Benfratelli Hospital, 90127 Palermo, Italy

**Keywords:** Parkinson’s disease, mild cognitive impairment, PD-MCI, non-invasive brain stimulation, tRNS, DLPFC, executive functions

## Abstract

Background/Objectives: Mild cognitive impairment (MCI) is common in Parkinson’s disease (PD) and often precedes dementia. Non-invasive brain stimulation (NIBS) techniques such as transcranial random noise stimulation (tRNS) targeting dorsolateral prefrontal cortex (DLPFC) may offer additional benefits for cognitive and motor functions in PD-MCI patients. Methods: Using a randomized, double-blind, cross-over study, participants with PD-MCI completed two stimulation sessions (real vs. sham) 7 days apart. Cognitive and motor outcomes (MoCA, FAB, FAS, MDS-UPDRS motor) were assessed pre- and post-stimulation; stimulation was administered “online” during executive training. Scores before and after the sessions have been compared, as well as their variations between the two groups. Results: Ten subjects were in the study. Patients undergoing real tRNS showed improvements in global cognition and executive functioning compared to those undergoing sham stimulation, as demonstrated by significant increase in MoCA and FAB scores. In contrast, the motor examination showed no significant differences. Conclusions: This preliminary study showed that a single session of DLPFC-tRNS stimulation produced domain-specific cognitive benefits in PD-MCI patients. Studies with multiple stimulation sessions and larger samples are needed to confirm the effect of this non-pharmacological therapeutic option in PD-MCI.

## 1. Introduction

Cognitive decline is recognized as a common feature of Parkinson’s disease (PD) and encompasses all stages of the dementia continuum, ranging from subjective cognitive decline to mild cognitive impairment (MCI) and dementia [1,2,3,4]. Parkinson’s disease dementia (PDD) affects approximately 30% of patients with PD [2], and a three to five times increased risk of PDD was described in PD patients with MCI at baseline [5,6]. Given the significant impact of cognitive impairment in PD on both patients and caregivers, early identification of individuals at high risk of cognitive decline and the associated risk factors are of considerable clinical and prognostic importance [2,7]. Therefore, the research focuses on the identification of pre-clinical (and potentially reversible) stages of dementia, such as MCI, defined as an objective deficit in one or more cognitive domains (memory, attention, language, or executive functions) while functional autonomy in daily activities is maintained. In 2012, a task force of the Movement Disorder Society (MDS) developed a set of standardized diagnostic criteria for PD-MCI, intended for use in both clinical and research settings [8]. Using these criteria, PD-MCI has been identified even in newly diagnosed patients, with a prevalence ranging from 14.8% to 42.5% [3,9,10].

In recent years, non-invasive brain stimulation (NIBS) has attracted growing interest as a non-pharmacological approach to ameliorate cognitive decline in patients with neurodegenerative disorders. By enhancing neuronal plasticity, techniques such as transcranial magnetic stimulation (TMS) and transcranial direct current stimulation (tDCS) have shown promising results as adjunct treatments to improve cognitive outcomes in MCI, dementia, and PD [11,12,13].

TDCS is a NIBS technique that applies low-intensity electric currents to specific brain regions to modulate neuronal excitability [14,15]. Main advantages of tDCS include its painless nature, the ability to selectively target specific cortical regions, and its small size, which allows for home use. Indeed, a systematic review and meta-analysis [16] of self-administered home-based tDCS applications revealed high levels of patients’ satisfaction, with clinically meaningful reductions in chronic pain intensity at the end of the interventions. These results suggest that NIBS techniques can be safely and conveniently applied at home, increasing the spread of neuromodulation therapies to different pathological areas.

Depending on the polarity, anodal currents increase cortical excitability, while cathodal ones decrease it [17]. Several studies on healthy subjects showed that tDCS can enhance various cognitive functions depending on the stimulated brain region. Clinical trials have also explored the rehabilitative potential of tDCS in neurological and psychiatric conditions. To date, several protocols have been applied to enhance performance in cognitive and motor tasks, as well as in the treatment of various neurological and psychiatric disorders [18].

Another non-invasive transcranial electrical stimulation protocol is represented by transcranial random noise stimulation (tRNS), which applies an alternating current with a randomly varying frequency, generated stochastically [19,20]. The phenomenon of stochastic resonance allows the membrane potential to fluctuate around the threshold without causing a constant hyperpolarization or depolarization, as occurs with tDCS depending on the electrode used. The fluctuations generated by a randomly varying current are relevant because some of them can synchronize with the physiological fluctuations occurring during synaptic activity, thereby facilitating the activation of neurons that would otherwise remain silent and increasing the system’s sensitivity. In terms of outcomes, tDCS produces a direct alteration of cortical excitability, causing either hyperpolarization or depolarization. In contrast, tRNS results in a summation between endogenous and exogenous neural fluctuations, leading to neuronal synchronization and enhanced plasticity [21,22].

Although tRNS may have greater potential than tDCS in the management of neurodegenerative diseases, few studies have investigated its cognitive effects in PD, focusing mainly on tDCS over the dorsolateral prefrontal cortex (DLPFC). Evidence regarding the efficacy of transcranial alternating current stimulation (tACS) or tRNS in PD shows that tRNS, with its balanced noise, does not drive cortical excitability in a single direction as tDCS does, but rather enhances the overall responsiveness of the circuit, preventing an increase in the neuronal asymmetry observed in PD. The aim of the present study is to evaluate the cognitive and, potentially, motor effects of a single session of tRNS applied over the DLPFC in patients with PD-MCI through the comparison of neuropsychological scores obtained before and after the stimulation. The DLPFC was targeted with the aim of achieving cognitive improvements related to executive functions, as well as a possible influence on motor skills, due to the anatomical contiguity with the motor cortex. Anatomical contiguity is relevant because stimulation through tRNS leads to a dispersion of the electric field across different tissues and neighboring areas, rather than confining it strictly to the stimulated site.

## 2. Materials and Methods

### 2.1. Study Design

This was a prospective crossover, double-blind, randomized, and sham-controlled study in subjects with PD-MCI who underwent tRNS applied to the DLPFC. The study was conducted in the outpatient clinics of the Dementia/Parkinson Center at the “Paolo Giaccone” University Hospital of Palermo, Italy. All participants provided their written informed consent prior to participating in the study, which was approved by the local Medical Ethics Committee (approval reference 03/2018, date of approval 14 March 2018) and conducted in accordance with the principles of the Declaration of Helsinki.

The study was divided into three sessions. The first consisted of a screening phase (baseline) to confirm the patient’s eligibility. The second and third sessions, held 7 days apart, consisted of cognitive and motor assessments performed before and after single sessions of tRNS, one active and one sham for each patient. The stimulator parameters were set by an experienced physician, who configured two distinct protocols, which were concealed from the experimenter and the patients, and applied in alternating order (first stimulation type chosen randomly in a balance manner) across the two stimulation sessions. For the first stimulation, computer software was used to generate a pseudo-random sequence of intervention (A or B), including a balanced number of the two. For the second stimulation, the hidden experimenter had to use the other setting. The tRNS machine was set in the “blind mode”, so that no details of the stimulation were displayed apart from the remaining stimulation time and intervention code (i.e., A or B). Further details of this protocol have been described in a previous study of the same research group [23]. A third researcher (statistician) was involved in the data analysis, obtaining the results for intervention A or B. Only at the end of the analysis, the person who had blinded the protocols revealed which was the real and the sham one.

### 2.2. Inclusion and Exclusion Criteria

Inclusion criteria:

- At least 5 years of formal education;
- Age between 50 and 80 years;
- Diagnosis of PD, clinically at stage I–III according to the Hoehn and Yahr classification [24];
- Stable antiparkinsonian medication regimen for at least 4 weeks;
- Presence of MCI [8];
- Ability to provide informed consent.

Exclusion criteria:

- Contraindications to tRNS (e.g., scalp skin diseases, history of neurosurgical interventions);
- Neurological comorbidities (excluding PD-MCI);
- Presence of major depressive disorder, using a cutoff of ≥15 on the Hospital Anxiety and Depression Scale [25];
- Use of psychotropic drugs (e.g., antidepressants, anxiolytics, or neuroleptics) or cognitive enhancers.

### 2.3. Study Protocol Structure

#### 2.3.1. Baseline Evaluation

During the first session, patients were screened for MCI using Addenbrooke’s Cognitive Examination-Revised (ACE-R) test [26]. Cut-off scores for the diagnosis MCI in absence of impairments in functional autonomy (activities of daily living and instrumental activities of daily living) using ACE-R were set as follows [27]:

- Attention and Orientation ≤ 14.73;
- Memory ≤ 14.47;
- Verbal Fluency ≤ 6.01;
- Language ≤ 18.83;
- Visuospatial Abilities ≤ 10.73.

#### 2.3.2. First Stimulation

The first stimulation session began once all study inclusion criteria had been verified. This session was divided into three phases as follows:

- Pre-stimulation (T0)

In this phase, the patient underwent a brief battery of neuropsychological tests, including the Montreal Cognitive Assessment (MoCA), Italian version 8.1, a scale for evaluating global cognitive functioning in individuals with MCI [28]; the Frontal Assessment Battery (FAB) [29] and the Phonemic Verbal Fluency Test (FAS) to assess executive functions [30]. Motor assessment was conducted using the motor section of the Unified Parkinson’s Disease Rating Scale (MDS-UPDRS) [31]. The total score for each test was recorded. In addition, separate scores for MoCA subitems (i.e., memory, visuospatial, attention, language, abstraction, orientation, and naming) were noted as well.

b. Stimulation

The stimulation phase consisted of a single tRNS session delivered over the left DLPFC, administered as either active or sham depending on the assigned protocol.

The device used was the BrainStim EMS stimulator. This battery-powered unit generated a current conveyed via leads to the electrodes. Surface electrodes (4.5 × 4.5 cm) were covered with 7 × 6.5 cm sponge pads soaked in saline solution to reduce impedance. Electrode placement was guided by an EEG cap; in accordance with the International 10–20 system, the stimulating anode was positioned at F3 (left DLPFC, being the area where there is most evidence of cognitive enhancement by NIBS [32]), while the cathode was located on an extracephalic point (right shoulder). Electrodes were secured in place with elastic straps.

Parameters applied for active (real) stimulation were as follows:

- Intensity: 1.5 mA;
- Frequency range: 100–600 Hz;
- Stimulation duration: 900 s (15 min).

Parameters applied for placebo (sham) stimulation were as follows:

- Intensity: 1.5 mA;
- Frequency range: 100–600 Hz;
- Stimulation duration: 900 s (15 min); however, current was delivered only during the first and last 30 s to give participants the impression of being stimulated, as cutaneous sensations are typically perceived mainly at these times during the active protocol.

**Figure 1:**
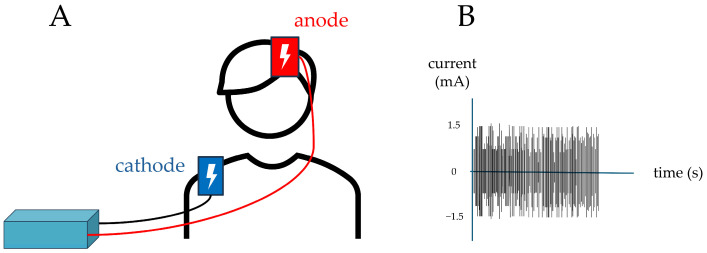
On the left (**A**) a representation of the montage of the electrodes; on the right (**B**) a representation of the tRNS real stimulation paradigm.

The stimulation procedure was conducted “online”. During this procedure, concurrently with the stimulation, active or sham, the patient underwent cognitive training targeting executive functions associated with the stimulated region, namely the DLPFC. The neuropsychological tests administered during the training were Raven’s Colored Progressive Matrices [33] and Backward Digit Span [34]. Figure 1 represents a schematization of the real stimulation procedure.

c. Post-Stimulation (T1)

In the post-stimulation phase, the same tests administered during the pre-stimulation phase were performed to enable comparison of the results obtained before and after stimulation: MoCA, FAB, FAS, MDS-UPDRS Motor Examination. To prevent a learning (practice) effect, an alternate MoCA form was administered, specifically the Italian version 8.2.

#### 2.3.3. Second Stimulation

In the third session, the patient underwent the second tRNS stimulation over the left DLPFC, scheduled 7 days after the previous session. During this second stimulation session, the operator changed the (blinded) protocol on the device to stimulate either real or sham, depending on the protocol used in the first stimulation session.

The phases of the second stimulation session mirrored those of the first: Pre-stimulation (T2), Stimulation, and Post-stimulation (T3). The tests administered in this session were again MoCA, FAB, FAS, and the UPDRS motor section. To minimize practice effects, different versions of the Montreal Cognitive Assessment were employed: Italian version 8.3 during the pre-stimulation phase (T2) and Italian version 8.1 during the post-stimulation phase (T3).

### 2.4. Statistical Analysis

Statistical analyses were performed using Jamovi (Version 2.6). Looking singularly at the real and sham groups, cognitive and clinical data pre- and post-stimulation have been evaluated according to normal distribution test (Shapiro) using paired Student’s *t* test or Wilcoxon’s rank test. Moreover, the variation in the score of each outcome measure has been calculated in each group. Then, the different variations (real vs. sham) have been compared using an independent samples *t* test or Mann–Whitney U test. Quantitative variables have been reported as mean or median with standard deviation (SD) with their range when needed.

## 3. Results

### 3.1. Study Population

The study included 10 participants (8 males, 2 females; mean age 67 years, range 55–77). The mean disease duration was 7.3 years (range 1–13). The average daily levodopa equivalent dose was 518.7 mg (median 500 mg), with a range from 0 to 885 mg/day. Table 1 summarizes the characteristics of the included population.

### 3.2. Effect of tRNS Session

Looking at the outcome measures before and after stimulation, significant changes have been observed in the real group for the FAB (pre 14.7 ± 2.6 vs. post 15.7 ± 2.7, *p* = 0.022) and for the MoCA total score (pre 22.0 ± 3.8 vs. post 23.9 ± 4.2, *p* = 0.018). In addition, a variation just above significance has been observed in the visuospatial MoCA subitem in the sham group (pre 3.0 ± 1.0 vs. post 3.6 ± 1.2, *p* = 0.048). Table 2 shows the comparisons between the two time points in the two groups.

**Figure 2:**
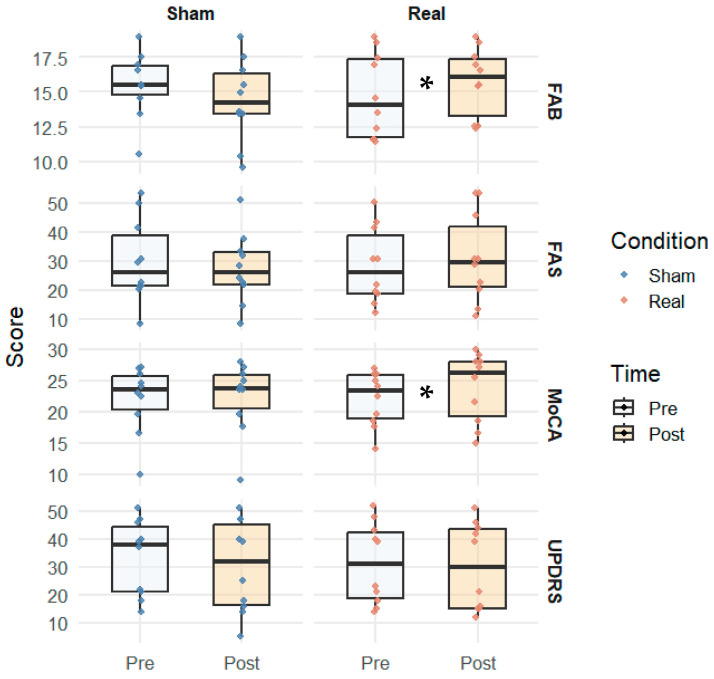
Box plots showing the interquartile range of pre- and post-stimulation results of the main outcome measures of the two groups. Abbreviations: FAB = Frontal Assessment Battery; FAS = Phonemic Verbal Fluency Test; MoCA = Montreal Cognitive Assessment; UPDRS = Unified Parkinson’s Disease Rating Scale. * *p* < 0.05.

**Figure 3:**
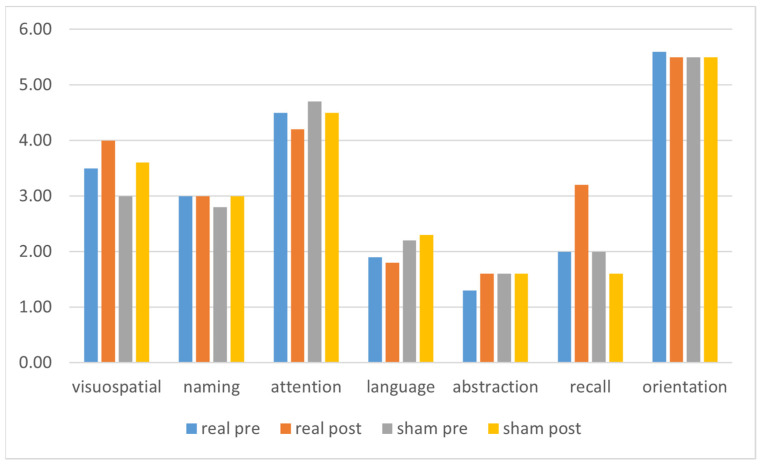
Graphical representation of the MoCA subitems before and after stimulation in the two different groups. Abbreviations: MoCA = Montreal Cognitive Assessment.

Figure 2 presents a graphical representation of the results of the main outcome measures (i.e., FAB, FAS, MoCA, UPDRS total scores), while Figure 3 shows a graphical representation of the MoCA subitems results.

**Figure 4:**
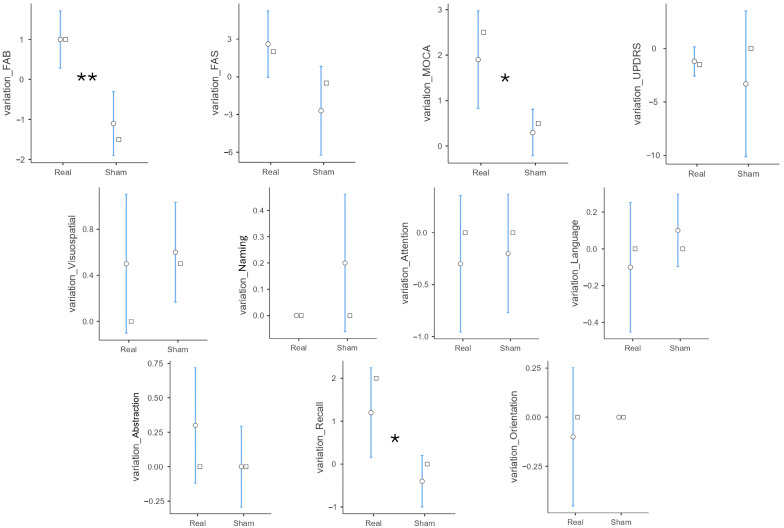
Graphical representation of the variations in the analyzed outcome measures. The white dot represents the mean with the 95% confidence interval, while the white square represents the median value. ** *p* < 0.01, * *p* < 0.05. Further details on the analyses can be found within the text.

Moreover, analyzing the variations in tests scores, a significant was found for some of the items, such as MoCA total score (real 1.90 ± 1.73 vs. sham 0.30 ± 0.82, *p* = 0.017), MoCA verbal memory recall subitem (real 1.20 ± 1.69 vs. sham −0.40 ± 0.97, *p* = 0.018), and FAB (real 1.00 ± 1.15 vs. sham −1.10 ± 1.29, *p* = 0.001), again demonstrating a positive effect of the tRNS in the real group compared to sham. Table 3 shows the tests scores variations and comparison between the two groups, while Figure 4 presents a graphical representation.

## 4. Discussion

The presented study suggests a potential effect of a single session of tRNS applied to the DLPFC in improving cognitive performance in PD patients with MCI. Conversely, the data from this study did not demonstrate any effect of tRNS stimulation on the motor performance of PD-MCI patients.

The improvement in cognitive performance in PD-MCI patients after DLPFC stimulation observed in the present study is consistent with data obtained from previous studies that used single sessions of NIBS in patients with PD. Indeed, single-session studies have reported significant improvements in working memory, verbal fluency, and divided attention in PD patients following anodal tDCS (atDCS) over the DLPFC [35,36,37]. Similarly, a multi-session protocol by Doruk et al. [38] demonstrated enhanced executive functioning (Trail Making Test B) in patients with PD with effects lasting even at one-month follow-up. However, not all results have been consistent: a single-session study by Lau et al. [39], applying anodal tDCS over the left DLPFC, did not find significant changes in visual working memory or go/no-go task performance. However, none of the aforementioned studies included patients presuming cognitive impairment in the spectrum of PD non-motor symptoms, while standardized criteria for PD-MCI were not used.

In our sample, real tRNS over the DLPFC significantly improved executive functions evaluated by FAB. This is a cognitive domain that is often impaired in patients with PD, whether they show MCI and/or PDD [40]. Therefore, improving patients’ performance by NIBS technique in addition to cognitive training could represent a breakthrough in the management of cognitive decline in PD patients. In addition, tRNS showed a general improvement in global cognition as demonstrated by a significant increase in the MoCA total score. This should come as no surprise, as the MoCA also includes a subitem evaluating executive functioning. The connections between DLPFC and hippocampus [41] may explain the positive influence of tRNS stimulation on long-term episodic memory, as demonstrated by an increase in the MoCA memory recall subitem. Overall, studies conducted with NIBS techniques in patients with PD and cognitive decline seem to suggest a positive effect on cognitive functions when applied to the DLPFC; however, given the differences in study protocols and sample characteristics evaluated in the various studies, further data are needed, conducted on large case series and with multiple session protocols, in order to draw definitive conclusions.

An innovative aspect of our study regarded the stimulation choice. In fact, most studies use tDCS with its anodal or cathodal stimulation. Conversely, we decided to use tRNS to take advantage of its influence on brain circuitry oscillations. However, studies including a comparison of multiple techniques could be useful in the future to establish which setting could be better in different situations. A strength of our study was the evaluation of both cognitive and motor performances in patients with diagnosed PD-MCI according to Level I Litvan’s et al. criteria [8]. Only a few studies have addressed the effects of tDCS on both motor and cognitive domains in patients with PD-MCI diagnosed according to the current criteria. Manenti et al. [42] showed that a two-week atDCS protocol over the DLPFC, combined with physical therapy, led to improvements in both motor and cognitive function in PD-MCI patients diagnosed using Level I MDS criteria. Similarly, a single-session study by Adenzato et al. [43] applying atDCS over the medial frontal cortex in patients with PD-MCI diagnosed according to Level I MDS diagnostic criteria, reported enhanced theory of mind abilities. Conversely, studies that used the more comprehensive Level II MDS diagnostic criteria have yielded mixed results. Biundo et al. demonstrated the long-term effectiveness of repeated sessions (4 sessions per week for 4 weeks) of left DLPFC anodal tDCS together with cognitive training [44]. To obtain a personalized approach, Del Felice et al. decided to study the EEG power spectra of PD patients and to use tACS to try to normalize the increased brain oscillations. This EEG-guided repeated protocol for 2 weeks induced an improvement of motor symptoms (bradykinesia) and of cognitive performances (MoCA score) [45].

In the sample studied in the present research, we did not observe any significant improvement in MDS-UPDRS scores. Similar inconsistencies have already been reported in the literature, where the effects of NIBS on motor outcomes in PD remain heterogeneous. These discrepancies may be due to differences in stimulation parameters and protocol duration (single vs. repetitive sessions), as well as the absence of concomitant physical therapy, given that our online protocol specifically targeted cognitive functions. Indeed, the plasticity-enhancing effects of NIBS are generally optimized when combined with task-specific functional training. In addition, it is possible that the DLPFC modulation may improve complex motor aspects that could be studied with more sensitive measures than UPDRS. Indeed, Wong et al. demonstrated how repeated sessions of anodal tDCS over left DLPFC lead to an improvement in the ability to walk while performing another activity (i.e., dual task walking—DTW) [46]. This is an interesting aspect that shows us the potential of NIBS techniques and their applications to such complex aspects of the disease. Finally, another possibility could be to test whether the effects of tRNS differ across disease stages, since the degree of neurodegeneration and cortical plasticity may influence responsiveness to stimulation.

Another aspect to consider is the possible influence of NIBS techniques on neuroinflammation. Several data suggest that neuroinflammation plays a relevant role in PD [47]. On the other hand, some researchers demonstrated how tDCS can positively act reducing neuroinflammation biomarkers in different diseases such as strokes [48]. Thus, future research directions on NIBS for PD and PD-MCI may consider the influence on neuroinflammation biomarkers in these specific populations.

Although our study has some strengths (comprehensive motor and cognitive evaluations, use of Litvan’s diagnostic criteria for PD-MCI, and exclusion of patients with clinically relevant comorbid depressive symptoms), it also has some limitations that should be acknowledged. First, the small sample size limits the generalizability of our findings. Second, the single-session design and the lack of follow-up prevent us from drawing conclusions about the duration of the observed effects or their potential cumulative benefits with repeated stimulations. Third, although the cross-over design reduces interindividual variability, possible carry-over or learning effects cannot be entirely ruled out despite the 7-day washout period. Furthermore, the absence of concomitant physical therapy and the focus on cognitive rather than motor training may have limited potential impact on motor outcomes.

Future studies with larger samples, repeated-session protocols, and multimodal outcome measures are needed to confirm and extend these preliminary findings. Moreover, investigating additional cortical targets to enhance implicit learning mechanisms in PD-MCI patients may provide further therapeutic benefits.

## 5. Conclusions

The present study shows how online tRNS applied to the DLPFC is a non-invasive technique capable of improving cognitive functioning in PD-MCI patients. Particularly, as expected, executive functions showed the greatest improvement. However, even global cognition showed improvement, together with long-term verbal memory recall abilities. Larger samples and trials with repeated sessions are needed to confirm and extend such findings and to establish the correct stimulation parameters, which will hopefully lead to widespread use of this technique in home setting as well. Furthermore, in the near future, preliminary investigations conducted with advanced techniques such as quantitative electroencephalography or functional magnetic resonance imaging could help to design the best stimulation protocols.

## Figures and Tables

**Table 1:** Demographic and clinical characteristics.

Variable	Mean ± SD	Min–Max
Age (years)	67.0 ± 7.4	55–77
Disease duration (years)	7.3 ± 4.0	1–13
Levodopa dose (mg/day)	518.7 ± 298.3	0–885
Sex	8 Male (80%)/2 Female (20%)	—
Number of participants	10	—

**Table 2:** Comparisons of pre- and post-stimulation outcome measures for sham and real groups.

Test	Real Pre (M ± SD)	Real Post (M ± SD)	*p*-Value	Sham Pre (M ± SD)	Sham Post (M ± SD)	*p*-Value
FAB	14.7 (±2.6)	15.7 (±2.7)	**0.022**	15.5 (±2.3)	14.4 (±2.8)	0.057
FAS	28.4 (±12.0)	31.0 (±12.5)	0.087	30.0 (±10.0)	27.3 (±11.5)	0.176
MoCA total	22.0 (±3.8)	23.9 (±4.2)	**0.018**	22.0 (±3.5)	22.3 (±4.0)	0.299
Visuospatial	3.5 (±1.1)	4.0 (±1.0)	0.168	3.0 (±1.0)	3.6 (±1.2)	**0.048**
Naming	3.0 (±0.0)	3.0 (±0.0)	na	2.8 (±0.4)	3.0 (±0.0)	0.346
Attention	4.5 (±1.3)	4.2 (±1.5)	0.586	4.7 (±1.5)	4.5 (±1.6)	0.572
Language	1.9 (±0.9)	1.8 (±0.9)	0.773	2.2 (±0.8)	2.3 (±0.7)	1.000
Abstraction	1.3 (±0.8)	1.6 (±0.7)	0.233	1.6 (±0.7)	1.6 (±0.6)	1.000
Recall	2.0 (±1.2)	3.2 (±1.3)	0.061	2.0 (±1.0)	1.6 (±1.0)	0.265
Orientation	5.6 (±0.7)	5.5 (±0.8)	0.773	5.5 (±0.7)	5.5 (±0.6)	na
UPDRS (motor)	31.3 (±12.5)	30.1 (±13.2)	0.114	33.5 (±12.0)	30.2 (±13.0)	0.611

Bold values were considered statistically significant. Abbreviations: FAB = Frontal Assessment Battery; FAS = Phonemic Verbal Fluency Test; MoCA = Montreal Cognitive Assessment; na = not assessable due to zero variance in the data; UPDRS = Unified Parkinson’s Disease Rating Scale.

**Table 3:** Independent samples *t*-test: delta real vs. delta sham.

Parameter	Group	Mean	SD	*p*-Value
variation_FAB	Real	1.00	1.15	**0.001**
	Sham	−1.10	1.29	
variation_FAS	Real	2.60	4.27	0.087
	Sham	−2.70	5.70	
variation_MoCA	Real	1.90	1.73	**0.017**
	Sham	0.30	0.82	
variation_Visuospatial	Real	0.50	0.97	0.742
	Sham	0.60	0.70	
variation_Naming	Real	0.00	0.00	0.167
	Sham	0.20	0.42	
variation_Attention	Real	−0.30	1.06	1.000
	Sham	−0.20	0.92	
variation_Language	Real	−0.10	0.57	0.357
	Sham	0.10	0.32	
variation_Abstraction	Real	0.30	0.67	0.244
	Sham	0.00	0.47	
variation_Recall	Real	1.20	1.69	**0.018**
	Sham	−0.40	0.97	
variation_Orientation	Real	−0.10	0.57	0.584
	Sham	0.00	0.00	
variation_UPDRS	Real	−1.20	2.20	0.444
	Sham	−3.30	11.01	

Bold values were considered statistically significant. Abbreviations: FAB = Frontal Assessment Battery; FAS = Phonemic Verbal Fluency Test; MoCA = Montreal Cognitive Assessment; UPDRS = Unified Parkinson’s Disease Rating Scale.

## Data Availability

Data supporting the findings of this study are available from the corresponding author upon reasonable request due to privacy reasons.

## Abbreviations

MCI	Mild cognitive impairment
PD	Parkinson’s disease
MDS	Movement Disorder Society
NIBS	Non-invasive brain stimulation
tRNS	Transcranial random noise stimulation
DLPFC	Dorsolateral prefrontal cortex
PDD	Parkinson’s disease dementia
TMS	Transcranial magnetic stimulation
tACS	Transcranial alternating current stimulation (tACS)
ACE-R	Addenbrooke’s Cognitive Examination-Revised
MoCA	Montreal Cognitive Assessment
FAB	Frontal Assessment Battery
FAS	Phonemic Verbal Fluency Test
UPDRS	Unified Parkinson’s Disease Rating Scale
SD	Standard deviation
